# Bone stress fractures and injuries of the upper limb in athletes

**DOI:** 10.1007/s00264-026-06978-x

**Published:** 2026-08-08

**Authors:** Milos Bojovic, Elisabet Hagert

**Affiliations:** Aspetar Orthopaedic and Sports Medicine Hospital, Doha, Qatar

**Keywords:** Bone stress injury, Stress fracture, Upper limb, Sports medicine, Magnetic resonance imaging, Return to sport

## Abstract

**Purpose:**

Bone stress injuries of the upper limb are less common than those of the lower limb, but they can cause substantial diagnostic delay in athletes because symptoms are often subtle and non-specific. This narrative review summarizes clinically relevant mechanisms, risk factors, diagnostic challenges, anatomical patterns, management strategies, and return-to-sport considerations.

**Methods:**

This narrative review was developed from a targeted, non-systematic review of the current literature and clinical experience, with emphasis on sports that expose the shoulder, arm, forearm, wrist, and hand to repetitive loading. Particular attention was given to imaging features, regional presentations, and practical decision-making in athletic populations.

**Results:**

Upper-limb bone stress injuries occur when repetitive loading exceeds the capacity for skeletal adaptation, progressing from marrow oedema and periosteal reaction to cortical disruption if loading continues. Plain radiographs may be normal early, whereas magnetic resonance imaging is most useful for detecting early stress reactions. Injuries may affect the scapula, clavicle, humerus, ulna, radius, carpal bones, metacarpals, and phalanges. Management usually involves load modification, immobilization when appropriate, structured rehabilitation, and correction of biomechanical or training-related contributors. Surgery is reserved for selected displaced, non-union, or functionally limiting injuries.

**Conclusion:**

Recognition of upper-limb bone stress injuries requires a high index of suspicion. Individualized, multidisciplinary care is essential to prevent progression, guide safe return to sport, and reduce recurrence.

## Introduction

In high-level sport, clinical attention is usually drawn first to muscle strains, tendinopathies, and ligament injuries. Bone stress injuries (BSI) of the upper limb, however, may remain clinically quiet and therefore overlooked. Their presentation is often gradual and non-specific, in contrast to the more familiar lower-limb stress injuries such as tibial stress fractures in runners or navicular stress injuries in jumping athletes. As a result, upper-extremity BSI are still insufficiently recognized and are often diagnosed late or initially labelled as soft-tissue overload conditions [1–4]. This is particularly important because the upper limb is exposed to repeated high mechanical demands in throwing, gymnastics, weightlifting, racket sports, and other disciplines that require forceful gripping, axial loading, or overhead activity [5–8].

**Fig. 1:**
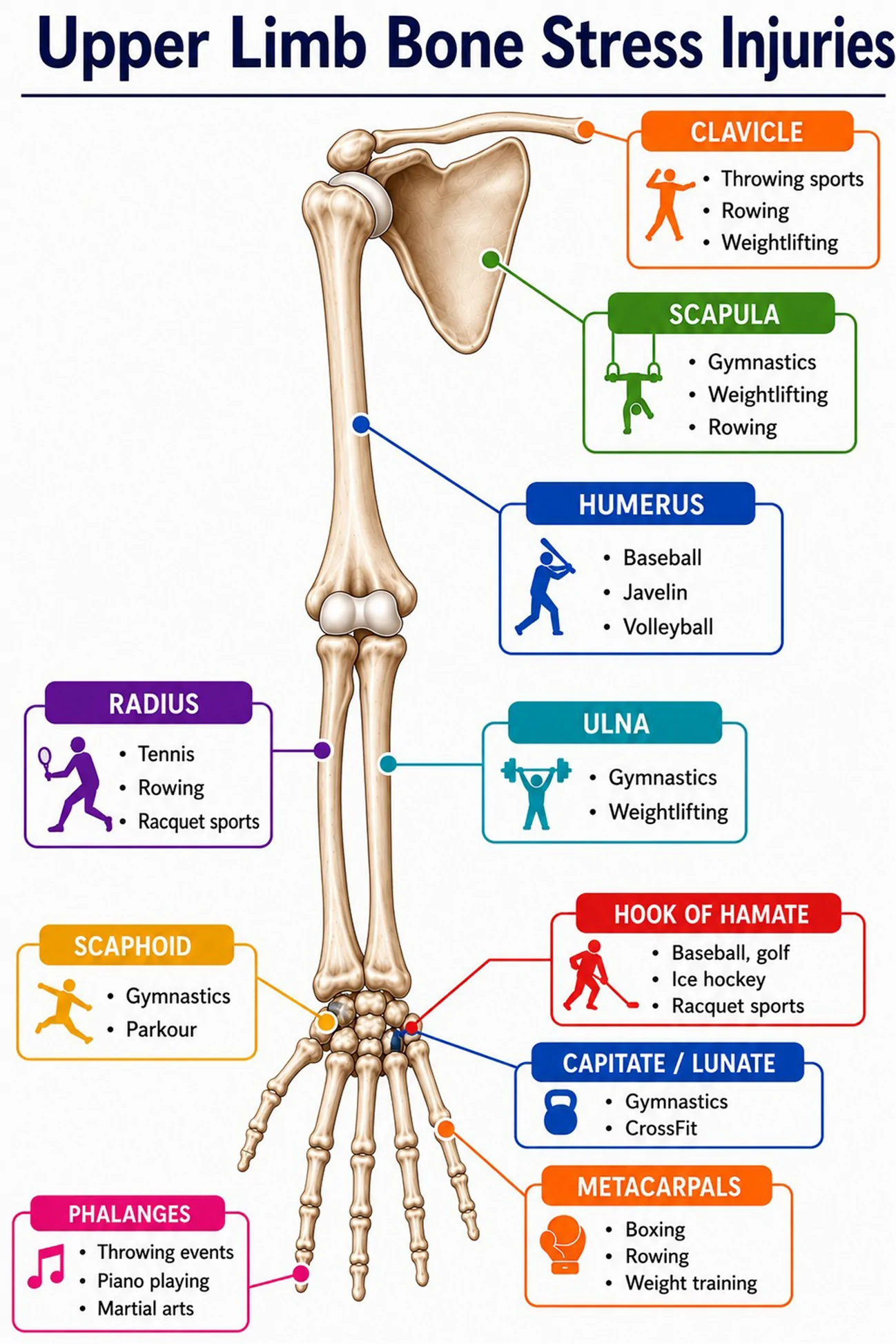
Schematic overview of upper-extremity bones that may be affected by bone stress injury

A practical message for clinicians is that no region of the upper limb should be considered immune to stress-related skeletal injury. Cases have been described from the distal clavicle in volleyball and rowing athletes, to the olecranon in throwing athletes, and the scaphoid or carpal bones in gymnasts and acrobatic athletes as shown in Fig. 1 [9–15]. Without an appropriately high level of clinical suspicion, these injuries can be confused with tendinopathy, joint irritation, inflammatory symptoms, or even pain behaviours without an identifiable structural explanation [13, 16].

This narrative review highlights the spectrum of BSI affecting the upper limb. Drawing on current evidence and clinical experience, it outlines mechanisms, risk factors, diagnostic pitfalls, regional presentations, management principles, and return-to-sport considerations. The central goal is to support earlier recognition of these injuries before a reversible stress reaction progresses to a complete structural failure.

### Pathophysiology and risk factors of upper-limb BSI

BSI develops when the adaptive capacity of bone is exceeded by repetitive mechanical loading. Under normal circumstances, bone remodels in response to stress, but when loading is excessive, recovery is inadequate, or both occur together, microscopic damage accumulates faster than it can be repaired [3, 17]. Early in the process, the injury may be limited to a stress reaction, with marrow oedema and periosteal response that are usually visible only on sensitive imaging such as magnetic resonance imaging (MRI) [18–20]. Continued loading may then lead to a stress fracture, characterized by a visible fracture line and varying degrees of cortical disruption [21].

At the biological level, the injury reflects an imbalance between bone formation and bone resorption. Mechanical loading normally stimulates osteoblast-mediated bone formation and can increase local bone strength. When repeated loading is not matched by sufficient recovery, osteoclastic activity may predominate, producing resorption, microdamage progression, and reduced structural resistance [22–25]. This mismatch is especially relevant in adolescent athletes, where rapid skeletal growth, high training loads, and insufficient intake of key nutrients such as calcium and vitamin D may compromise bone mineralization and overall skeletal resilience [26].

Specific anatomical sites in the upper limb are exposed to characteristic mechanical stresses [27]. In overhead athletes, humeral shaft stress injuries are often related to repetitive torsional and bending loads generated during the throwing or striking motion [15, 16, 28, 29]. Ulnar stress fractures are more often seen in sports with repeated pronation-supination, impact, or axial loading, including fast-pitch softball and kendo, where the cumulative mechanical demand may exceed the capacity for local bone adaptation [30–33].

Children and adolescents require particular attention because their physes are still open and their skeletal physiology is changing rapidly [34, 35]. Repetitive stress in this population may present as Little League shoulder, with widening of the proximal humeral physis after repetitive throwing, or gymnast’s wrist, where repeated axial loading and hyperextension cause stress changes around the distal radial physis [8, 14, 36].

The development of BSI is influenced by both intrinsic and extrinsic factors [4, 8, 37]. Intrinsic contributors include anatomical alignment, local biomechanics, muscle imbalance, endocrine status, and nutritional adequacy. Relative Energy Deficiency in Sport (RED-S), particularly in female athletes but not limited to them, may increase risk through impaired energy availability, hormonal disturbance, and negative effects on bone density regulation [3]. Extrinsic contributors include abrupt changes in training volume or intensity, insufficient rest, technical errors, inappropriate equipment, and repetitive exposure to sport-specific loads without adequate periodization [5, 7, 10].

For clinicians assessing athletes with upper-limb pain, an understanding of these mechanisms and risk factors is essential. Early identification allows timely modification of training, correction of technique or equipment-related contributors, assessment of nutritional status, and prevention of progression from a stress reaction to a more established fracture.

### Diagnostic challenges in upper-limb BSI

Upper-limb BSI are diagnostically challenging because the early clinical picture is often subtle. Athletes may report poorly localized pain that appears gradually, worsens with loading, and initially improves with rest. Because these symptoms resemble common soft-tissue disorders, the severity of the underlying bone injury can be underestimated [8, 38]. Delayed recognition may prolong symptoms and, in some cases, contribute to malunion, delayed union, persistent pain, or inability to return to sport [16, 28, 33].

The physical examination is frequently non-specific. Local tenderness and mild swelling may be present, but strength and range of motion can be preserved or only minimally affected [4]. Provocative maneuvers may help localize symptoms in selected cases, such as a humeral squeeze test in suspected humeral involvement, but these tests are not sufficiently sensitive or specific to exclude adjacent soft-tissue pathology [29].

Plain radiographs are usually obtained first, yet they are often normal in the early stages because they do not reliably demonstrate marrow oedema or subtle periosteal reaction [39, 40]. A normal radiograph can therefore falsely reassure both clinician and athlete, delaying further investigation and appropriate load management [9, 41].

Magnetic resonance imaging (MRI) is the most useful modality for early diagnosis, as it can demonstrate marrow oedema, periosteal reaction, early cortical change, and subtle fracture lines before abnormalities are obvious on radiographs [3, 17, 18, 20]. Bone scintigraphy and single-photon emission computed tomography (SPECT) may also assist in difficult cases or where MRI is unavailable, although they provide less detailed anatomical information than MRI and may be less accessible in routine practice [42].

Awareness is particularly important in less typical sites, such as the scapula, hamate, scaphoid, capitate, or lunate, where rarity and atypical symptoms may lead clinicians away from a bone diagnosis [10–13, 43]. In the paediatric and adolescent population, the presentation may be different again because stress injury can involve the physis rather than the mature cortex [34–36]. Accurate diagnosis therefore depends on careful history taking, recognition of sport-specific loading patterns, early use of appropriate imaging, and close collaboration between sports physicians, radiologists, physiotherapists, coaches, and, where needed, surgeons [2, 36].

### Regional anatomy-based review of upper-limb BSI

Table 1 summarizes common sports, mechanisms, and clinical clues for BSI across the main anatomical regions of the upper limb.

**Table 1:** Common upper-limb BSI by anatomical region, sport, mechanism, and clinical presentation

Region	Common sports	Typical mechanism	Clinical clues
*Scapula*	Gymnastics, weightlifting, rowing	Repetitive overhead loading and axial compression	Posterior shoulder pain; reduced tolerance for overhead activity
*Clavicle*	Throwing sports, rowing, weightlifting	Repeated compression or traction across the shoulder girdle	Focal distal clavicle pain; pain during cross-body adduction
*Humerus*	Baseball, javelin, volleyball	Torsional load during throwing, serving, or hitting	Mid-arm pain during throwing; possible ulnar nerve-type symptoms
*Ulna*	Gymnastics, weightlifting, tennis	Axial load combined with wrist or elbow hyperextension, wrist ulnar deviation and rotation	Dorsal forearm pain; symptoms with resisted wrist extension: pro/supination
*Radius*	Tennis, rowing, racquet sports, gymnastics	Repeated pronation-supination and axial loading	Pain with forearm rotation or wrist loading; distal radial tenderness
*Scaphoid*	Gymnastics, parkour	Axial load in wrist hyperextension	Snuffbox tenderness; reduced grip strength
*Capitate/Lunate*	Gymnastics, crossfit	Repeated compression and shear across the wrist	Deep wrist pain, particularly during loading
*Hook of hamate*	Baseball, golf, ice hockey, racquet sports	Repetitive impact from bat, racket, stick, or club handle	Deep volar-ulnar hand pain; pain while gripping equipment
*Metacarpals*	Boxing, rowing, weight training	Punching forces or repeated heavy gripping	Dorsal hand pain, worse with punching or gripping
*Phalanges*	Throwing events, piano playing, martial arts	Repetitive fine-motor loading or direct impact	Focal finger pain, aggravated by impact or repetitive tasks

### Scapula and clavicle

Scapular stress fractures are uncommon but should be considered in athletes exposed to repetitive overhead activity or axial loading, especially in gymnastics, weightlifting, and rowing [9–11]. Reported sites include the scapular body, spine, and acromion [27]. Athletes typically describe vague posterior shoulder pain that increases with overhead use, together with localized tenderness. Because radiographic findings can be subtle, diagnosis often requires MRI or computed tomography (CT). Treatment is usually non-operative and includes rest from the provoking activity, load modification, and physiotherapy aimed at strengthening, scapular control, and correction of contributing biomechanical deficits [5, 41].

Stress injury of the distal clavicle is most relevant in athletes whose sport produces repeated compression or traction around the shoulder girdle, including throwers, rowers, and weightlifters. The clinical picture is usually focal distal clavicular pain and tenderness, often reproduced by cross-body adduction [44, 45]. Early radiographs may not be diagnostic, so MRI or CT may be required. Most cases recover with conservative care, including temporary unloading, activity modification, and progressive rehabilitation [3].

### Humerus

**Fig. 2:**
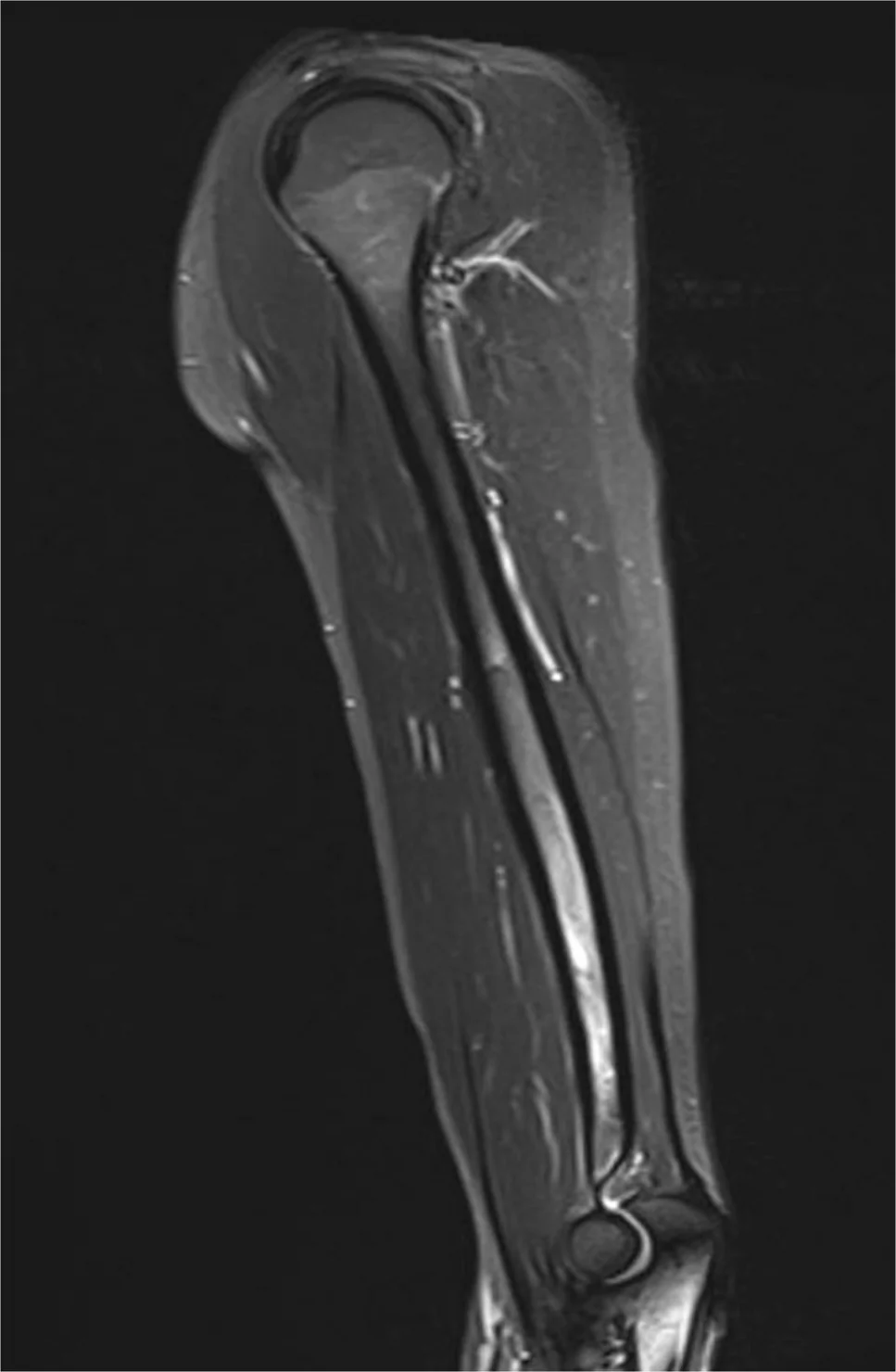
MRI demonstrating diffuse bone marrow oedema in the distal humeral diaphysis with mild periosteal reaction along the anteromedial distal humerus, consistent with bone stress injury

Humeral BSI can involve the proximal, middle, or distal thirds of the bone, depending on the dominant loading pattern [16, 28]. A well-known example is the so-called thrower’s fracture, a spiral stress fracture associated with repetitive torsional stress in throwing athletes, particularly baseball pitchers [29]. The diagnosis may be complicated by symptoms that resemble neural irritation, and ulnar neuropathy can occasionally mimic or coexist with humeral stress injury [15]. Athletes typically report mid-arm pain during throwing or hitting, sometimes accompanied by paraesthesia or numbness. MRI is the preferred imaging modality [39], as shown in Fig. 2. Treatment is usually conservative, beginning with rest and followed by a structured rehabilitation programme and gradual return-to-throwing or return-to-hitting progression [3, 40].

### Ulna and radius

**Fig. 3:**
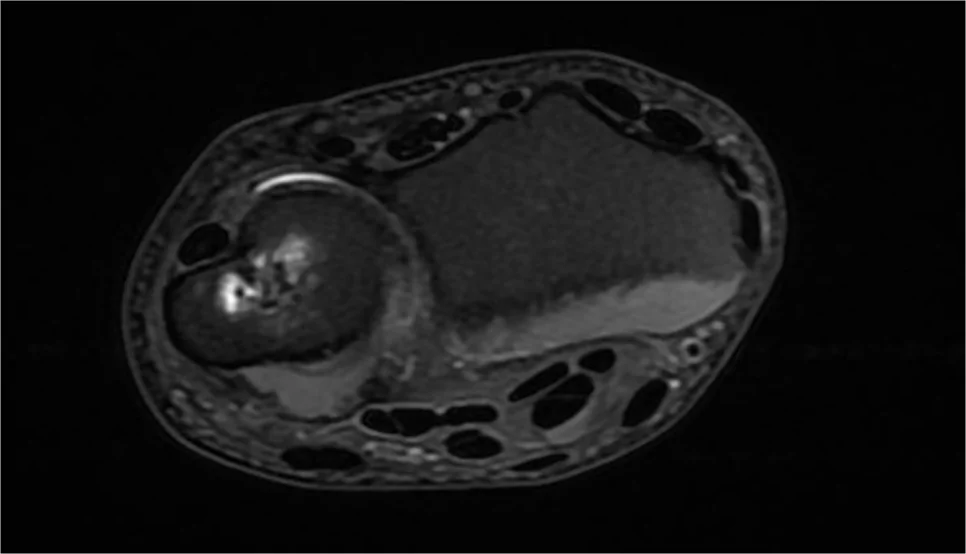
Focal bone marrow oedema involving the distal ulna

Ulnar shaft stress fractures have been reported in gymnasts, weightlifters, softball pitchers, and other athletes exposed to repetitive axial loading, forearm rotation, or hyperextension forces [30–32]. Symptoms usually include dorsal forearm pain that worsens with activity, resisted wrist extension, or forearm rotation. Bone stress reactions without a visible fracture line may also involve the ulnar head and distal ulna (Fig. 3.) in tennis/racket sports, leading to pain in rotation and wrist ulnar deviation. Because early radiographic changes can be absent, MRI and, in selected cases, bone scintigraphy are useful for early diagnosis [46]. Most cases heal with immobilization, activity modification, and gradual reloading [3].

Radial stress injuries, particularly around the distal metaphysis, have been described in tennis players, rowers, and athletes exposed to repeated pronation-supination or axial wrist loading [5]. Clinical findings include tenderness over the distal radius and pain during wrist or forearm motion. MRI is important for early detection because plain radiographs may be normal or only subtly abnormal [19, 20, 41]. Stress injury of the radial head is rare but may occur in sports requiring repetitive forearm rotation; diagnosis often depends on advanced imaging such as MRI or SPECT [19].

### Bones of the wrist and hand

Scaphoid stress fractures occur particularly in gymnasts, acrobatic dancers, and athletes who repeatedly load the wrist in hyperextension [12]. Typical findings include anatomical snuffbox pain on palpation, pain on wrist loading and reduced grip strength. MRI or CT is essential for timely diagnosis. Management usually begins with immobilization in non-displaced fractures, while surgery may be considered when diagnosis is delayed or healing is complicated, as in proximal pole scaphoid fractures [6, 21].

Stress fractures of the capitate and lunate are rare but relevant in gymnastics, CrossFit, and other sports involving repeated compression and shear through the wrist. Athletes usually report deep wrist pain provoked by loading activities [7, 47]. MRI provides the clearest diagnostic information, and conservative management is often successful [3].

**Fig. 4:**
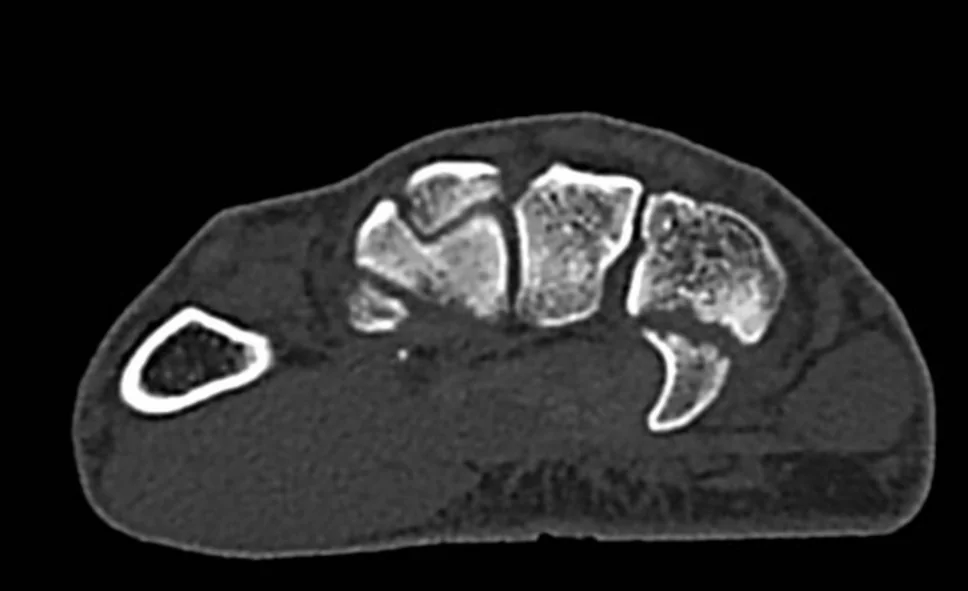
Hook of hamate, non-union

Hook of hamate stress injury should be suspected in athletes using a bat, racket, stick, or club, including baseball, tennis, ice hockey, and golf. Repetitive contact between the handle and the ulnovolar aspect of the hand may cause microtrauma that progresses to a stress fracture of the hamate hook [48]. These fractures are frequently missed on standard radiographs and often require MRI or CT (Fig. 4.) for confirmation. Early diagnosis is important because this region is close to the deep motor branch of the ulnar nerve and the flexor tendons to the ring and little fingers, creating risk for nerve compression or tendon attrition if the injury is not recognized.

Metacarpal stress fractures are most often described in boxing, rowing, and weight training. Pain is typically dorsal and worsens with heavy gripping, punching, or sport-specific loading [8]. MRI may be required because radiographic findings can be subtle. Rest and immobilization remain the mainstays of treatment. Phalangeal stress injuries are uncommon but have been reported in climbers, throwers, musicians, and martial arts athletes [49, 50]. Diagnosis is based on compatible clinical features supported by imaging, and conservative treatment is usually effective [3].

### Management strategies for upper-limb BSI

#### Conservative management

Non-operative care is the first-line approach for most upper-limb BSI. The immediate priority is to reduce or stop the activity responsible for repetitive skeletal loading, allowing bone remodeling and repair to occur before the injury progresses. Immobilization with a splint, brace, or cast may be appropriate when a clear stress fracture is present or when pain persists with daily activities, as it reduces mechanical stress and supports healing [8, 36].

Rehabilitation should follow the period of unloading and should be structured rather than simply symptom guided. Key elements include progressive loading, restoration of range of motion, strengthening of relevant kinetic-chain and upper-limb muscles, and correction of biomechanical deficits. Neuromuscular control, scapular and trunk contribution, forearm strength, grip function, and sport-specific movement patterns may all need to be addressed, depending on the injury location and sport demands [3].

#### Surgical intervention

Although the majority of upper-limb BSI heal without surgery, operative treatment is required in selected cases [51]. Indications include displacement, non-union, failure of adequate conservative management, structural compromise, or persistent functional limitation despite appropriate rehabilitation [52]. Surgical management commonly involves internal fixation with screws or plates, aiming to restore stability, improve alignment, promote union, and in some cases allow a more predictable return to sport [6, 8, 36].

#### Monitoring recovery

Recovery should be monitored using a combination of clinical, functional, and imaging-based criteria [53]. Clinically, resolution of pain at rest, tenderness on palpation, and pain during progressive loading are important markers. Functionally, strength, range of motion, grip measures, throwing or hitting tolerance, and sport-specific tests help determine readiness for progression [37]. Imaging, particularly follow-up MRI where clinically indicated, can document improvement in marrow oedema and cortical restoration [20, 54].

A multidisciplinary model is often the safest strategy. Sports physicians, radiologists, physiotherapists, strength and conditioning staff, coaches, and orthopaedic surgeons should align the diagnosis, loading plan, imaging interpretation, and return-to-sport decision. This approach reduces the risk of recurrence and supports a more reliable return to performance [55].

### Return-to-sport considerations for upper-limb BSI

#### Criteria for return to sport

Return-to-sport decisions after upper-limb BSI should not be based on time alone. A multidimensional assessment is required, incorporating symptoms, examination findings, imaging, and functional capacity. The primary clinical requirement is complete absence of pain at rest and during progressively demanding sport-specific tasks [3]. Persistent symptoms suggest ongoing stress reaction or incomplete healing and should prompt further load modification or reassessment.

Radiological healing may be important, particularly in higher-risk anatomical sites or where the initial injury included a clear fracture line. CT and MRI can demonstrate resolution of marrow oedema, reduction of periosteal reaction, restoration of cortical continuity, and remodeling [20, 39]. Functional criteria should include objective assessment of strength, mobility, endurance, kinetic-chain control, and sport-specific performance to confirm that the athlete can tolerate the demands of training and competition.

#### Phases of reintegration into sports activities

Return to sport is best managed through staged progression [56]. The first phase focuses on pain-free restoration of joint range of motion and baseline muscular capacity. Loading is then reintroduced gradually, with careful attention to symptoms during and after activity.

The next phase involves modified sport participation at reduced volume and intensity. For throwers or hitters, this may include controlled drills with limited distance, frequency, speed, or total repetitions [57, 58]. As tolerance improves, the athlete progresses to intermediate sport-specific tasks, with incremental increases in intensity and complexity. Interval throwing or hitting programmes are useful examples, provided they are individualized and symptoms are monitored closely [59].

The final phase is unrestricted participation. Full return should only occur after the athlete remains symptom-free through progressive challenges and has met clinical, functional, and, where appropriate, imaging criteria. Table 2 presents a practical phased model for rehabilitation and return to sport after upper-limb BSI.

**Table 2:** Phased rehabilitation and return-to-sport progression following upper-limb BSI

Phase	Key activities	Criteria for progression
*Phase 1: Initial rehabilitation*	Restore range of motion and begin basic strengthening	Pain-free basic movements
*Phase 2: Modified sport participation*	Introduce controlled, low-impact sport-specific activity at reduced volume and intensity	No symptoms during low-intensity sport-specific loading
*Phase 3: Intermediate progression*	Increase sport-specific intensity, complexity, and volume in a graded manner	Intermediate drills completed without symptom recurrence
*Phase 4: Full sport reintegration*	Return to unrestricted full-intensity sport-specific tasks	Functional testing passed; radiological confirmation when required

#### Prevention of recurrence

Prevention requires identification and modification of the factors that produced the injury. Training errors, rapid workload progression, technical deficits, and local biomechanical contributors should be addressed before full return. Sport-specific technique analysis and targeted coaching may reduce repeated stress at vulnerable sites [1, 60].

Training plans should include progressive increases in volume and intensity, sufficient recovery periods, and appropriate periodization across the season. Bone health should also be optimized through adequate energy availability and sufficient calcium and vitamin D intake, particularly in adolescents and athletes with risk factors for relative energy deficiency in sport (RED-S) [26].

### Limitations of the review

This narrative review has several limitations. It was not based on a preregistered systematic search strategy or a formal risk-of-bias assessment. In addition, the literature specific to upper-limb bone stress injuries is limited and consists largely of case reports, case series, and narrative reviews, with some concepts extrapolated from research on lower-limb bone stress injuries. The heterogeneity of anatomical sites, sporting demands, imaging approaches, and reported treatment strategies limits direct comparison across studies and prevents firm conclusions regarding incidence, optimal management, and return-to-sport timelines. Accordingly, the present review should be interpreted as a clinically oriented narrative synthesis rather than a formal systematic review.

## Conclusion

Upper-limb BSI are less common than lower-limb stress injuries, but they represent an important and often under-recognized clinical problem in sports medicine. Their symptoms are frequently subtle, their anatomical distribution is broad, and their early imaging findings may be missed if clinicians rely only on plain radiographs. For these reasons, early clinical suspicion, careful history taking, and timely advanced imaging are central to diagnosis.

Effective management requires more than rest. Load modification, appropriate immobilization when indicated, structured rehabilitation, correction of sport-specific risk factors, and individualized return-to-sport progression are important components of management. Education of athletes, coaches, and healthcare professionals may reduce diagnostic delays and prevent complications related to missed or undertreated injuries.

Future research should consider refining the classification systems for upper-limb BSI according to anatomical site, sport-specific biomechanics, and imaging features. Further work is also needed on biological therapies, prevention strategies, and objective return-to-sport criteria. Improved awareness and dedicated research may lead to earlier diagnosis, more precise treatment, fewer recurrences, and safer sustained athletic performance.

## Data Availability

No datasets were generated or analysed during the current study.
